# Supplementary material for: qsmR encoding an IclR-family transcriptional factor is a core pathogenic determinant of Burkholderia glumae beyond the acyl-homoserine lactone-mediated quorum-sensing system
Source: PLoS Pathog. 2024 Oct 3;20(10):e1011862. doi: 10.1371/journal.ppat.1011862 (PMC11478832; doi:10.1371/journal.ppat.1011862)
Supplement: S1 Text — Table A. Genome features and statistics of the three Burkholderia glumae strains used in this study. Table B. Pairwise comparison of the average nucleotide identity (ANI) between B. glumae strains. Table C. Pairwise comparison of virulence-related gene products in the amino acid sequence identity between B. glumae strains. Fig A. Phylogenetic analysis of selected rice-associated B. glumae strains based on the aligned proteins and coding DNA from single-copy genes using RaxML v.8 program. Fig B. A schematic depiction of the tofI/tofM/tofR QS gene cluster deleted in the ΔtofI-R derivatives of 336gr-1 and 411gr-6. Black arrows indicate the primer set to confirm the gene deletion by PCR (the agarose gel image on right side). Fig C. The production of the purple pigment, violacein, by the biosensor Chromobacterium violaceum CV026 (McClean et al. 1997)[19] dependent on the acyl homoserine lactone (AHL) QS signal molecules from each strain of B. glumae. Fig D. The production of the AHL QS signal molecule of B. glumae by the ΔqsmR and ΔtofI-R or ΔtofI derivatives of 336gr-1 and 257sh-1. Fig E. Comparison of the structures of the QsmR proteins between the virulent strains (336gr-1, 411gr-6, and BGR1, purple) and the avirulent strain (257sh-1, green). Fig F. Restoration of toxoflavin production in 257ΔtofI-R by a qsmR clone carrying the virulent allele, pqsmR-1. The photo was taken 24 h after inoculation and following incubation at 37°C. (DOCX) [file ppat.1011862.s001.docx]

Table A. Genome features and statistics of the three *Burkholderia glumae* strains used in this study (Lee et al, 2021)[16].

| **Features** | ***B. glumae* strains** | | |
| --- | --- | --- | --- |
|  | **336gr-1** | **257sh-1** | **411gr-6** |
| Chromosome | 2 | 2 | 2 |
| Plasmid | 3 | 2 | 2 |
| NCBI Accession | CP023203-7 | CP035900-3 | CP021157-60 |
| Size (bp) | 6,879,338 | 6,691,685 | 6,747,595 |
| GC Content | 68.1 | 68.2 | 68.3 |
| N50 | 3.60 | 3.54 | 3.60 |
| Number of CDS | 6715 | 6531 | 6818 |
| Number of RNAs | 92 | 81 | 92 |

Figure A. Phylogenetic analysis of selected rice-associated *B. glumae* strains based on the aligned proteins and coding DNA from single-copy genes using RaxML v.8 program.

Table B. Pairwise comparison of the average nucleotide identity (ANI) between *B. glumae* strains.

| ***B. glumae* strains** | **ANI (%)​** |
| --- | --- |
| 257sh-1 vs. 336gr-1**3gr-1** | 99.45 |
| 257sh-1 vs. 411gr-6 | 99.92 |
| 336gr-1 vs. 411gr-6 | 99.47 |

Table C. Pairwise comparison of virulence-related gene products in the amino acid sequence identity between *B. glumae* strains

| **Category** | **Description** | **Name** | **336gr-1_257sh-1** | **336gr-1_411gr-6** | **257sh-1_411gr-6** |
| --- | --- | --- | --- | --- | --- |
| T2SS | Type II secretion system protein C | GspC | 100 | 100 | 100 |
| T2SS | Type II secretion system protein D | GspD | 99.62 | 99.62 | 100 |
| T2SS | Type II secretion system ATPase GspE | GspE | 100 | 100 | 100 |
| T2SS | Type II secretion system inner membrane protein GspF | GspF | 100 | 100 | 100 |
| T2SS | Type II secretion system major pseudopilin GspG | GspG | 100 | 100 | 100 |
| T2SS | GspH/FimT family pseudopilin | GspH | 100 | 100 | 100 |
| T2SS | Type II secretion system minor pseudopilin GspI | GspI | 100 | 100 | 100 |
| T2SS | prepilin-type N-terminal cleavage/methylation domain-containing protein | GspJ | 100 | 100 | 100 |
| T2SS | Type II secretion system minor pseudopilin GspK | GspK | 100 | 100 | 100 |
| T2SS | Type II secretion system protein GspL | GspL | 100 | 100 | 100 |
| T2SS | Type II secretion system protein M | GspM | 100 | 100 | 100 |
| T2SS | type II secretion system protein N | GspN | 100 | 100 | 100 |
| T3SS | Type III secretion protein HrpB7 | HrpB7 | 99.43 | 99.43 | 100 |
| T3SS | Type III secretion protein HrpB2 | HrpB2 | 100 | 100 | 100 |
| T3SS | Type III secretion protein, HrpB1 | HrpB1 | 100 | 100 | 100 |
| T3SS | HrpB4 protein | HrpB4 | 100 | 100 | 100 |
| T3SS | HpaP protein; Type III secretion protein (YscP) | HpaP | 99.55 | 99.55 | 100 |
| T3SS | Type III secretion outer membrane pore forming protein (YscC, MxiD, HrcC, InvG) | HrcC | 99.83 | 99.83 | 100 |
| T3SS | Type III secretion inner membrane protein (YscT, HrcT, SpaR, EscT, EpaR1, homologous to flagellar export components) | HrcT | 99.64 | 99.64 | 100 |
| T3SS | Type III secretion inner membrane channel protein (LcrD, HrcV, EscV, SsaV) | HrcV | 99.57 | 99.57 | 100 |
| T3SS | Type III secretion inner membrane protein (YscR, SpaR, HrcR, EscR, homologous to flagellar export components) | HrcR | 100 | 100 | 100 |
| T3SS | Type III secretion inner membrane protein (YscS, homologous to flagellar export components) | HrcS | 100 | 100 | 100 |
| T3SS | HrpD6 protein | HrpD6 | 100 | 100 | 100 |
| T3SS | Type III secretion HpaB protein | HpaB | 100 | 100 | 100 |
| T3SS | Type III secretion cytoplasmic ATP synthase (EC 3.6.3.14, YscN, SpaL, MxiB, HrcN, EscN) | HrcN | 99.78 | 99.78 | 100 |
| T3SS | Type III secretion bridge between inner and outermembrane lipoprotein (YscJ, HrcJ, EscJ, PscJ) | HrcJ | 100 | 100 | 100 |
| Flp/Tad pilus-encoding gene cluster | Flp pilus assembly protein TadB | TadB | 99.41 | 99.7 | 99.7 |
| Flp/Tad pilus-encoding gene cluster | Flp pilus assembly protein RcpC/CpaB | CpAB | 99.68 | 99.68 | 100 |
| Flp/Tad pilus-encoding gene cluster | Type II/IV secretion system protein TadC, associated with Flp pilus assembly | TadC | 99.39 | 99.39 | 100 |
| Flp/Tad pilus-encoding gene cluster | Type II/IV secretion system ATPase TadZ/CpaE, associated with Flp pilus assembly | TadZ/CpaE | 99.76 | 99.76 | 100 |
| Flp/Tad pilus-encoding gene cluster | Type II/IV secretion system secretin RcpA/CpaC, associated with Flp pilus assembly | RcpA/CpaC | 99.37 | 99.37 | 100 |
| Flp/Tad pilus-encoding gene cluster | Flp pilus assembly protein, pilin Flp | Flp | 100 | 100 | 100 |
| Flp/Tad pilus-encoding gene cluster | Type II/IV secretion system ATP hydrolase TadA/VirB11/CpaF, TadA subfamily | TadA | 99.55 | 99.55 | 100 |
| Type VII (Chaperone/Usher pathway, CU) | type 1 fimbriae major subunit FimA | FimA | 100 | 100 | 100 |
| T6SS | T6SS component TssA (ImpA) | TssA | 100 | 100 | 100 |
| T6SS | T6SS component TssB (ImpB/VipA) | TssB | 100 | 100 | 100 |
| T6SS | T6SS component TssC (ImpC/VipB) | TssC | 100 | 100 | 100 |
| T6SS | T6SS lysozyme-like component TssE | TssE | 100 | 100 | 100 |
| T6SS | T6SS component TssF (ImpG/VasA) | TssF | 100 | 99.84 | 99.84 |
| T6SS | T6SS component TssG (ImpH/VasB) | TssG | 99.73 | 99.73 | 100 |
| T6SS | T6SS AAA+ chaperone ClpV (TssH) | TssH | 100 | 100 | 100 |
| T6SS | T6SS secretion lipoprotein TssJ (VasD) | TssJ | 100 | 100 | 100 |
| T6SS | T6SS component TssK (ImpJ/VasE) | TssK | 100 | 100 | 100 |
| T6SS | T6SS outer membrane component TssL (ImpK/VasF) | TssL | 100 | 100 | 100 |
| T6SS | T6SS component TssM (IcmF/VasK) | TssM | 100 | 100 | 100 |
| Predicted effector protein | 3-demethylubiquinone-9 3-methyltransferase | VOC family virulence protein | 100 | 100 | 100 |
| Predicted effector protein | Pyridoxine 4-dehydrogenase PhxI (EC 1.1.1.65) | PhxI | 98.99 | 98.99 | 100 |
| Predicted effector protein | Lysophospholipase L1 and related esterases | Tep-I | 99.77 | 99.77 | 100 |
| Lipase | Lipoyl synthase (EC 2.8.1.8) | LipA | 100 | 100 | 100 |
| Lipase | Octanoate-[acyl-carrier-protein]-protein-N-octanoyltransferase (EC 2.3.1.181) | LipB | 99.54 | 99.54 | 100 |
| Polygalacturonase | Glycoside hydrolase, family 28 | PehA | 100 | 100 | 100 |
| Polygalacturonase | hypothetical protein | PehB | 99.35 | 99.35 | 100 |
| Flagella | Flagellar transcriptional activator FlhD | FlhD | 100 | 100 | 100 |
| Flagella | Flagellar transcriptional activator FlhC | FlhC | 100 | 100 | 100 |
| Toxoflavin/EPS | Toxoflavin and eps regulator, TepR | TepR | 100 | 100 | 100 |
| IclR-family transcriptional factor | Toxoflavin and eps regulator | QsmR | 99.64 | 100 | 99.64 |
| Toxoflavin biosynthetic gene cluster | Methyltransferase ToxA | ToxA | 100 | 100 | 100 |
| Toxoflavin biosynthetic gene cluster | GTP cyclohydrolase II | ToxB | 100 | 100 | 100 |
| Toxoflavin biosynthetic gene cluster | WD-repeat-containing protein | ToxC | 100 | 100 | 100 |
| Toxoflavin biosynthetic gene cluster | Serine/threonine kinase | ToxD | 99.69 | 99.69 | 100 |
| Toxoflavin biosynthetic gene cluster | RibD | ToxE | 99.72 | 99.72 | 100 |
| Toxoflavin transport gene cluster | DMT family transporter | ToxF | 100 | 100 | 100 |
| Toxoflavin transport gene cluster | putative RND efflux membrane-fusion protein | ToxG | 100 | 100 | 100 |
| Toxoflavin transport gene cluster | putative RND efflux transporter | ToxH | 100 | 100 | 100 |
| Toxoflavin transport gene cluster | putative outer membrane protein OprM | ToxI | 99.61 | 99.62 | 99.61 |
| LuxR family transcriptional regulator | LuxR family transcriptional regulator ToxJ | ToxJ | 100 | 100 | 100 |
| LysR family transcriptional regulator | LysR type regulatory protein ToxR | ToxR | 100 | 100 | 100 |
| Protease | Serine metalloprotease | PrtA | 99.80 | 99.80 | 100 |
| Quorum-sensing regulatory protein | quorum-sensing regulatory protein | TofM | 99.33 | 99.33 | 100 |
| Quorum-sensing regulatory protein | LuxI‐family protein | TofI | 100 | 100 | 100 |
| Quorum-sensing regulatory protein | LuxR‐family protein | TofR | 99.58 | 99.58 | 100 |
| Two-component regulatory system | Two-component system sensor histidine kinase | PidS | 99.81 | 99.81 | 100 |
| Two-component regulatory system | Two-component transcriptional response regulator, OmpR family | PidR | 100 | 100 | 100 |
| Flagellar biosynthesis | Flagellar biosynthesis protein FliR | FliR | 100 | 100 | 100 |
| Flagellar biosynthesis | Flagellar biosynthesis protein FliQ | FliQ | 100 | 100 | 100 |
| Flagellar biosynthesis | Flagellar biosynthesis protein FliP | FliP | 100 | 100 | 100 |
| Flagellar biosynthesis | Flagellar biosynthesis protein FliO | FliO | 100 | 100 | 100 |
| Flagellar biosynthesis | Flagellar motor switch protein FliN | FliN | 100 | 100 | 100 |
| Flagellar biosynthesis | Flagellar motor switch protein FliM | FliM | 100 | 100 | 100 |
| Flagellar biosynthesis | Flagellar basal body-associated protein FliL | FliL | 100 | 100 | 100 |
| Transcriptional Regulator | Transcriptional regulator CidR, LysR family | CidR | 100 | 100 | 100 |
| Transcriptional Regulator | Transcriptional regulator, MarR family | MarR | 100 | 100 | 100 |
| Outer membrane factor (OMF) lipoprotein associated with MdtABC efflux system | Outer membrane factor (OMF) lipoprotein associated with MdtABC efflux system | Omp | 100 | 100 | 100 |
| Shikimate pathway | 3-phosphoshikimate 1-carboxyvinyltransferase (EC 2.5.1.19) | AroA | 99.77 | 99.77 | 100 |
| Shikimate pathway | 3-dehydroquinate synthase (EC 4.2.3.4) | AroB | 99.16 | 99.16 | 100 |


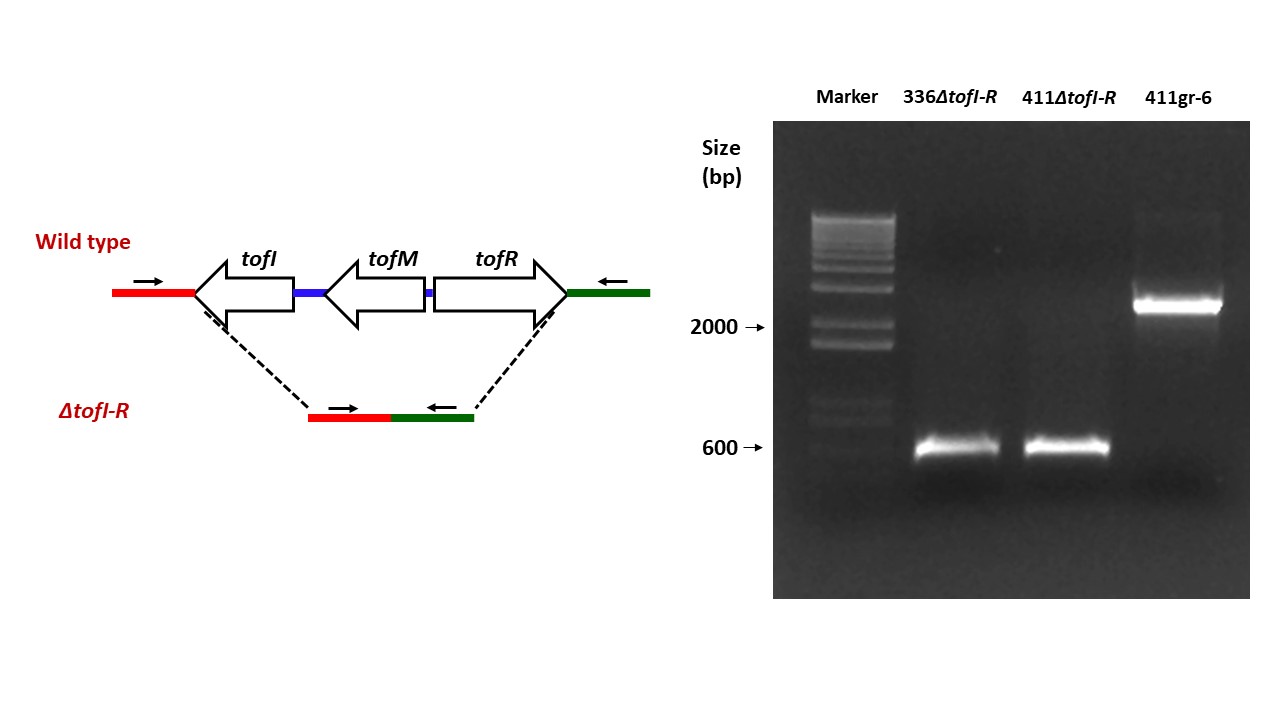


Figure B. A schematic depiction of the *tofI/tofM/tofR* QS gene cluster deleted in the *ΔtofI-R* derivatives of 336gr-1 and 411gr-6. Black arrows indicate the primer set to confirm the gene deletion by PCR (the agarose gel image on right side).


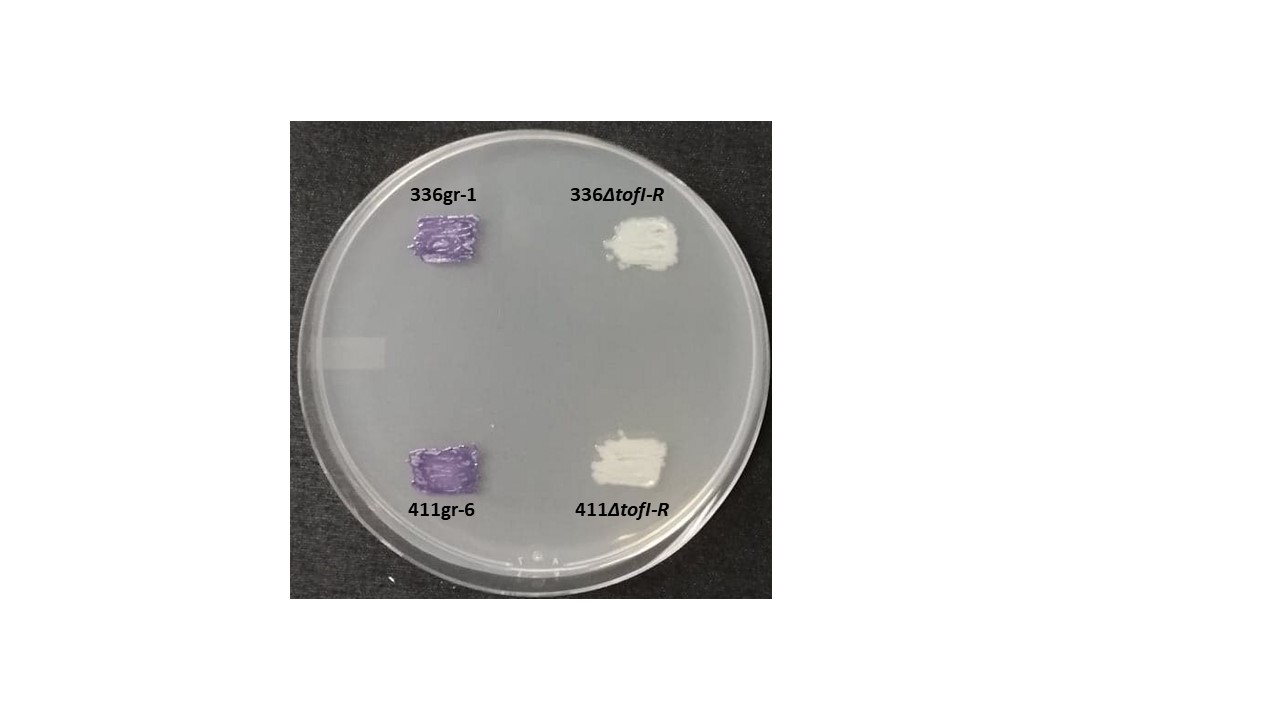


Figure C. The production of the purple pigment, violacein, by the biosensor *Chromobacterium violaceum* CV026 (McClean *et al.* 1997)[19] dependent on the acyl homoserine lactone (AHL) QS signal molecules from each strain of *B. glumae*.


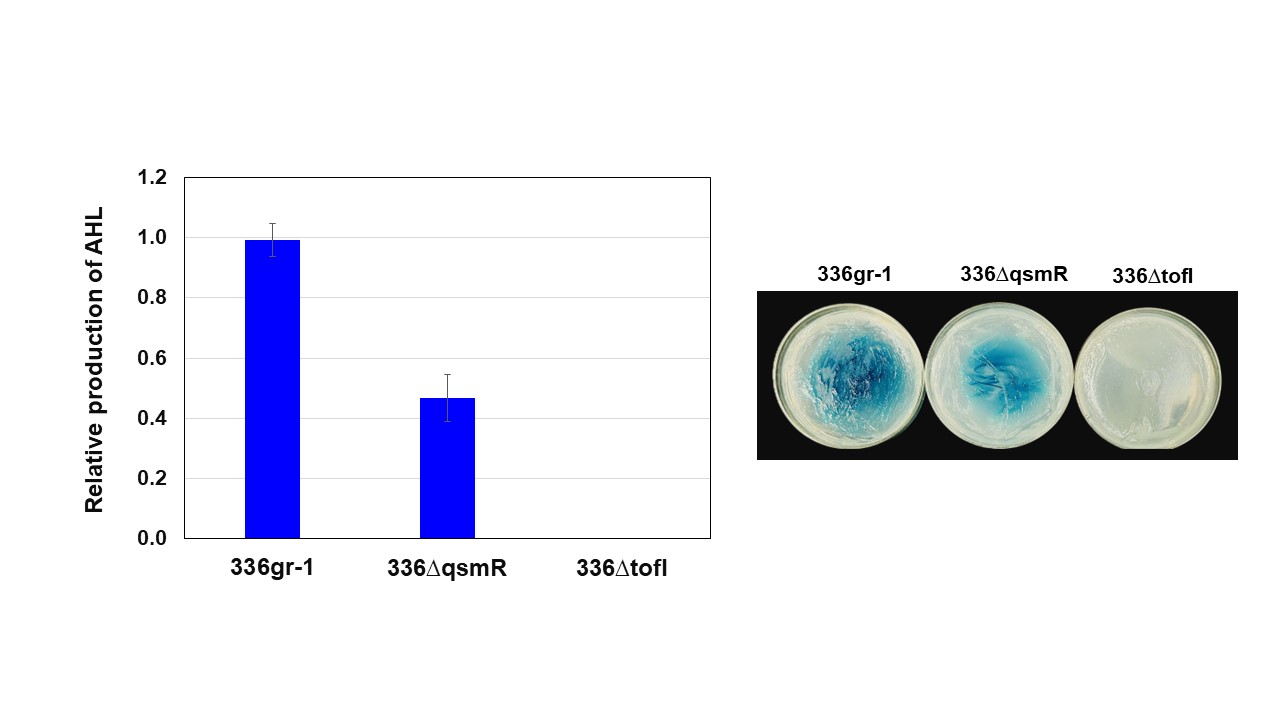


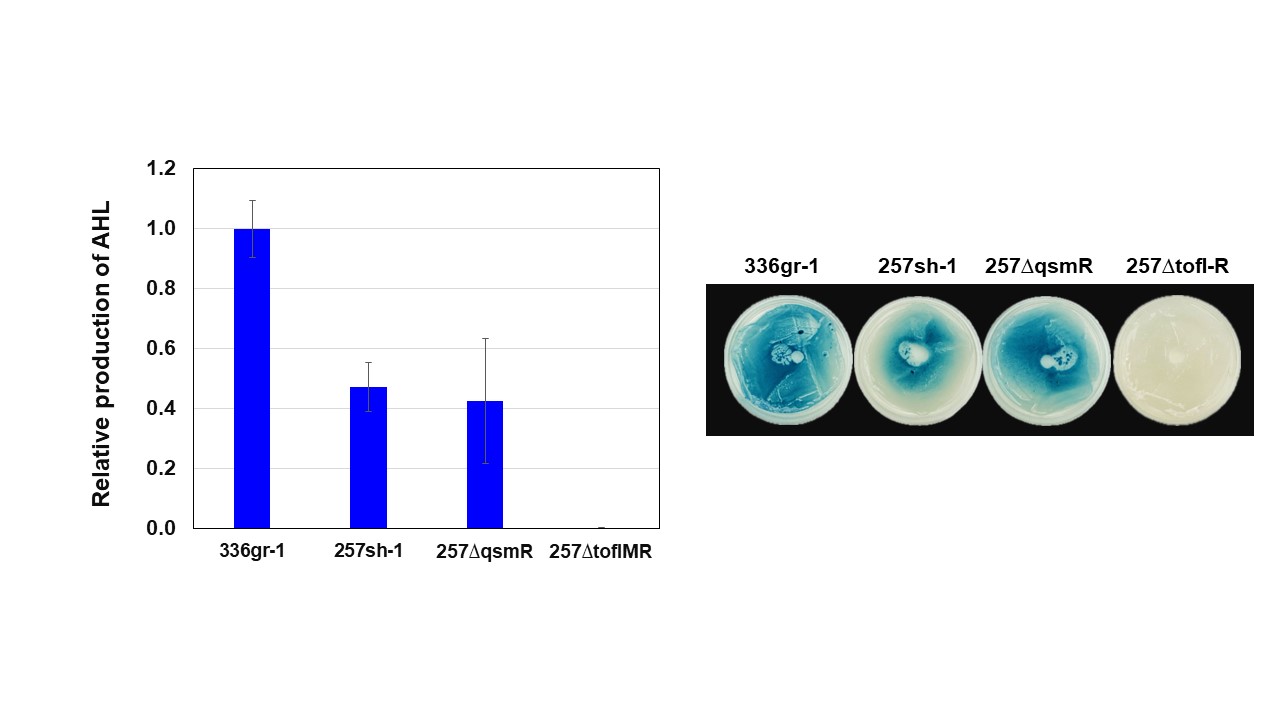


Figure D. The production of the AHL QS signal molecule of *B. glumae* by the *ΔqsmR* and *ΔtofI-R* or *ΔtofI* derivatives of 336gr-1 and 257sh-1.


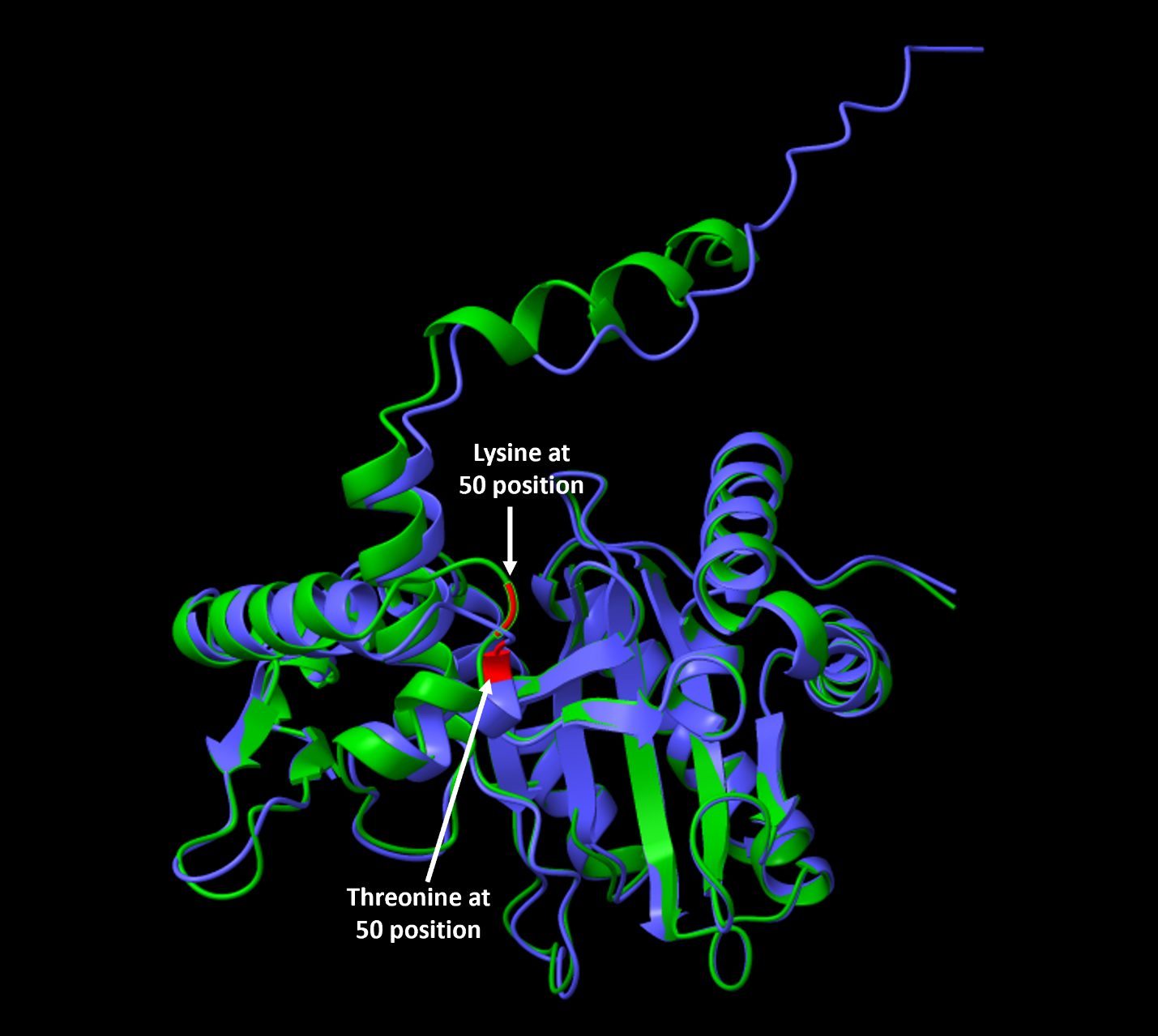


Figure E. Comparison of the structures of the QsmR proteins between the virulent strains (336gr-1, 411gr-6, and BGR1, purple) and the avirulent strain (257sh-1, green).


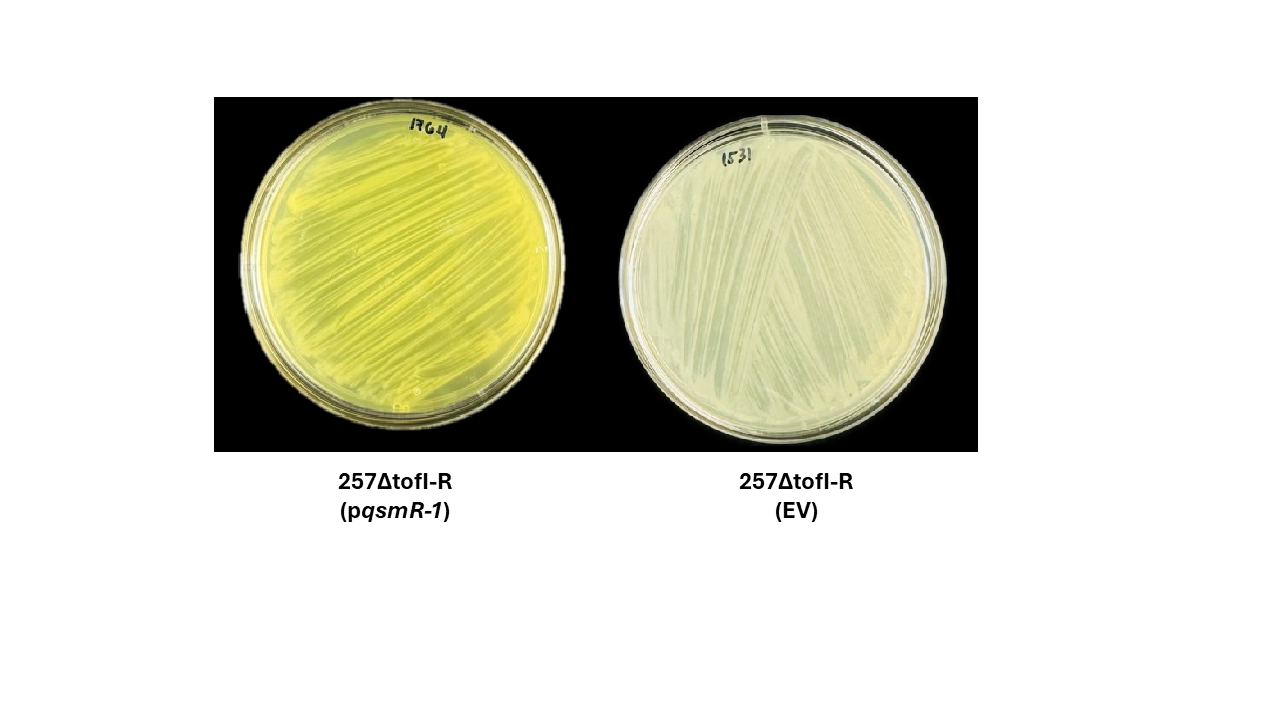


Figure F. Restoration of toxoflavin production in 257ΔtofI-R by a *qsmR* clone carrying the virulent allele, p*qsmR-1*. The photo was taken 24 h after inoculation and following incubation at 37°C.
