# Supplementary material for: qsmR encoding an IclR-family transcriptional factor is a core pathogenic determinant of Burkholderia glumae beyond the acyl-homoserine lactone-mediated quorum-sensing system
Source: PLoS Pathog. 2024 Oct 3;20(10):e1011862. doi: 10.1371/journal.ppat.1011862 (PMC11478832; doi:10.1371/journal.ppat.1011862)
Supplement: Attachment — Submitted filename: Respond_to_Reviewers_Comments.docx [file ppat.1011862.s003.docx]

Responses to Reviewers’Comments (In blue font)

**Part I - Summary**
Please use this section to discuss strengths/weaknesses of study, novelty/significance, general execution and scholarship.

Reviewer #1: In the manuscript titled "qsmR encoding an IclR-family transcriptional factor…," Lelis et al. conducted an extensive investigation into the role of the tof QS system and the qsmR regulator in the virulence of various Burkholderia glumae clones with distinct pathogenicity profiles. The authors found that the contribution of the tof system to virulence is strain-dependent, whereas qsmR is essential for pathogenesis across all tested strains. This reinforces prior findings by the same research group, solidifying qsmR as a key virulence regulator in B. glumae.

Moreover, the authors identified a single nucleotide polymorphism (SNP) in qsmR of the non-virulent strain B. glumae 257. Through complementation analyses, they demonstrated that the introduction of qsmR from other strains via plasmid successfully restored the pathogenicity of 257. This suggests that variations in qsmR may be a contributing factor to the loss of virulence in strain 257.

This study represents a substantial effort, and the results have the potential to significantly advance our understanding of B. glumae pathogenesis. In addition, the implications of SNP variants in qsmR for the pathogenicity of the bacterial population are particularly noteworthy, holding broader significance in the fields of pathogenomics and virulence regulation.

However, I have concerns regarding the study's methodology. Many experiments lack adequate controls, and certain analyses were inaccurately described (outlined in the "major revisions" section). These issues contribute to my reservations about the persuasiveness of the results. Consequently, I do not recommend the acceptance of this manuscript.

Reviewer #2: The manuscript PPATHOGENS-D-23-02079 is well-written and shows interesting results regarding mechanism of regulation of the pathogenicity on different Burkholderia glumae strains. The authors have demonstrated that the transcription regulator QsmR is a key factor on B. glumae virulence regulation since a residue mutation (T50K) in this protein has impaired the virulence. The observed results have indicated that QsmR can differently regulate the quorum-sensing genes tofI and tofR, which might dependent on the B. glumae strain analyzed. Though deletion of qsmR promoted the lost of HSL-C8 production on mutant strains, the mechanism which qsmR regulates the virulence in B. glumae has been not determined in this work. Following I have listed some points that may help to improve further some parts of this manuscript.

**Part II – Major Issues: Key Experiments Required for Acceptance**
Please use this section to detail the key new experiments or modifications of existing experiments that should be absolutely required to validate study conclusions.

Generally, there should be no more than 3 such required experiments or major modifications for a "Major Revision" recommendation. If more than 3 experiments are necessary to validate the study conclusions, then you are encouraged to recommend "Reject".

Reviewer #1: - The most exciting claim that the authors make in this study is that natural small variation in the coding sequence of a virulence regulator is the driving factor that dictates the aggressiveness of a pathogen. From an evolutionary standpoint, this result might have a major impact on the field of bacterial virulence regulation. However, I don't think that the authors did a good job of proving this point, and their key experiment lacked necessary controls. To address this issue, I suggest the authors consider one of the following options:

1. The authors used pBBR1MCS5 for "complementing" the 257 clone with 411/336qsmR and showed that the reintroduction restores virulence.
pBBR1MCS5 is a medium copy number plasmid (I believe it has ~20 copies), and its MCS is found after a lac promoter. Therefore, pBBR1MCS5-based complementation is a form of overexpression. With that in mind, the result observed by the authors in Fig. 9 can be caused by the overexpression of QsmR and not the variation in protein sequence. To address this, I suggest the authors clone qsmR from 257 into pBBR1MCS5, introduce it to 257, and repeat the experiment. I also strongly suggest adding a protein tag and confirming protein accumulation via Western blot. If introduction of qsmR257 does not turn 257 into a virulent strain, it will support the authors' hypothesis.

Response: Thank you for the good suggestions. To address the copy number issue, we constructed a 257-version of qsmR clone in pBBR1MC5 (named p*qsmR-A1*) and introduced it to 257sh-1. This 257sh-1 derivative carrying a 257-version of *qsmR* clone did not exhibit any maceration symptom and showed a basal level of toxoflavin like that carrying the vector plasmid alone. We added these results in the revised Fig. 9. The figure legend was revised accordingly, and the added information was reflected in the main text (lines 409 – 425 and 396-400) in the track-changed (TC) revision). Unfortunately, we could not have enough time to perform western blot analysis. This is mentioned in the discussion section of the revision (lines 548-549 in the TC revision).

2. A more elegant way to support the hypothesis is replacing the qsmR variant in the genome of 257 with qsmR from 411/336. This will address any issues that might arise from introducing a plasmid and potential overexpression since the gene will be expressed under its natural promoter, from its original genomic location, and in a single copy. I don't have experience with Burkholderia glumae, but as far as I understand, the sacB counter-selection system works well in this bacteria, and therefore, altering qsmR in the genome is doable.

Response: Thank you for the great suggestion. In fact, we had considered the suggested genetic test as a follow-up study after the publication of this study. Following this suggestion, however, we conducted the recommended experiments as a major additional work for this revision. For some reason, we had technical problems in introducing the *qsmR* clone of 336gr-1 in the suicide vector into 257sh-1. This was the main reason for the delayed submission of this revision.

Afterall, we could successfully impose swapping of *qsmR* between 257sh-1 and 336gr-1. We replaced the *qsmR* allele in 336gr-1 (virulent) with the *qsmR* having the 257sh-1 (avirulent) allele. As predicted, we found that the resultant mutants lost the ability to produce toxoflavin. In the same way, we swapped the *qsmR* allele in 257sh-1 (avirulent) with the 336gr-1 (virulent) allele. Again, the recombinants of 257sh-1 gained the 336gr-1 allele showed toxoflavin-positive phenotypes. These results prove that the SNP causing T50K substitution in QsmR confers the avirulent phenotype of 257sh-1. These results are added to the track-changed (TC) revision as Fig. 10 and mentioned in the text (lines 444 – 452, 427 -443, and 543 - 546 in the TC revision). The procedure for the allelic exchange is also added to the text (lines 774 – 784 in the TC revision).

- I disagree with the interpretation of the authors regarding the differential role of the tof system in the virulence of 411 and 336. In both cases, the tof system significantly contributes in a quantitative manner, but it is not essential, unlike qsmr (which appears to have a binary effect according to the study). 411 is more pathogenic and produces more toxoflavin and exopeptidase activity than 336. Therefore, the reduction in virulence caused by the disruption of tof is more apparent in 336. I actually think that this experiment supports that the differences in the virulence of these strains are unrelated to the tof system.

Response: Thank you very much for such an insightful opinion. We agree to this opinion so revised the relevant text as following: “The TofIR QS-deficient phenotypes of 336gr-1 and 411gr-6, as well as of 20 additional strains observed in this study, indicate that certain groups of *B. glumae* strains are less dependent on TofIR QS for virulence, implying the presence of additional regulatory/signaling pathway(s) for pathogenesis that is independent of TofIR QS.” (lines 625 – 628 in the TC revision), and “However, our results in this study demonstrate that the contribution of TofIR QS to the virulence of *B. glumae* is quantitatively variable among different strains.” (lines 693 – 695 in the TC revision).


- Outside of Fig. 9, the authors did not complement any of the mutants during the various analyses. Complementation is a crucial control in bacterial genetics-based studies and should be added to the experiments described in Fig. 2-7 (or at least some of them).

Response: We absolutely agree with this comment that any mutant phenotype of a gene should be confirmed by complementation test with a function copy of the gene (so called molecular Koch’s postulates). Unfortunately, however, we could not complement *qsmR-*deficient mutants of 336gr-1 and 411gr-6 by heterologous expression of the cloned *qsmR*. Only 257sh-1 showed gained virulence-related phenotypes by the *qsmR* clone, which included the maceration of onion scales and the toxoflavin production (Fig. 9 in the TC revision). This suggests that the feasibility of complementation for *qsmR* mutation varies among strains of *B. glumae*. We even observed that the heterologous expression of *qsmR* in the virulent wild type strain 336gr-1 reduced the toxoflavin production, which suggests that proper function of *qsmR* requires fine-tuned expression level. This issue is also addressed in the text (lines 566 – 574 in the TC text).

Nevertheless, Table 1 shows another important line of evidence for the essential function of *qsmR* in virulence. For the results shown in Table 1, we made deletion mutations of the *tofI/M/R* QS gene cluster and *qsmR* in 20 virulent strains of *B. glumae*. And the table reveals that *qsmR* is essential for virulence in all the 20 strains tested (Table 1). We addressed this point in the discussion section of the revision (lines 583 – 584 in the TC revision).

In case of Figures 2 and 3, which present the data of TofIR QS mutants, we did not include the complemented strains due to a similar reason, unstable or incosistent phenotype restorations by heterologous expression of the *tofI/tofM/tofR* QS gene cluster.

Reviewer #2: Major points:
1-In the text is mentioned that:“the transcription level of tofR, but not that of tofI, was significantly lower in 336ΔqsmR compared to its parent strain 336gr-1. However, in the other virulent strain 411gr-6, it was tofI, instead of tofR, that was suppressed by qsmR deletion (Figure 7B). Expression of toxA was completely suppressed in both 336ΔqsmR and 336ΔtofI-R, while it was partially (but significantly) suppressed in 411ΔtofI-R but completely suppressed in 411ΔqsmR (Figure 7B).” To strengthen these findings, I suggest to add exogenous C8-HSL in 411gr-6qmsR mutant strain culture and verify if it should recover toxA expression. It might indicate that qmsR acts regulating some point in C8-HSL biosynthesis in B. glumae strains.

Response: Thank you for the good suggestion. We conducted the suggested test and observed that addition of C8-HSL did not recover the toxoflavin production in the *qsmR* mutant of 336gr-1 or 411gr-6 (Fig. 7C and 7D in the TC revision). Moreover, we found that the production of AHL signal was not abolished but just reduced in 336gr-1, which was consistent with the qRT-PCR data showing partial reduction of *tofR* expression in the qsmR-deficient mutant. Remarkably, we also found that the avirulent strain 257sh-1 also produced AHL signal, but its production was not affected by *qsmR* deletion mutation (Fig. S4 in the revision). We added these results as a new Fig. S4 and addressed them in the text (lines 339 – 347, 351- 360, 846 – 848, and 939 - 952 in the TC revision). Instead, we removed the previous image in Fig. S4, which presented the abolishment of AHL production in *qsmR*-deficient mutants and modified the text according to the newly found results (lines 278 – 280 (deleted), 339 – 347, 667 – 670, and 676 – 681 (deleted), and 787 – 808 (deleted) in the TC revision). Based on these new data, we concluded that *qsmR* and *tof* QS are functionally independent, except that *qsmR* partially influences the *tofI-R* QS (lines 339 – 347, 667 – 670, and Figure 12 in the TC revision).

2- One important point that should be determined in this work is the action mechanism of the transcription regulator qmsR on virulence regulation of B. glumae. Based on gene expression analysis the authors should verify putative QmsR-binding site sequences and run some experiments with gene reporter fused to candidate promoter sequences and/or test the binding of the putative QmsR-binding site sequences and QmsR protein with gel shift assay. Is QmsR-binding site element present in different promoters TofR (for 336gr-1) and TofI (for 411gr-6) or in other factors that act in trans to regulate the virulence in B. glumae? Can QmsR-binding site be found on promoters of T3SS-coding genes or other essential genes for virulence in B. glumae?

Response: We are currently working on the establishment of an experimental system for biochemical assays, including gel shift assay, to dissect the functional mechanism of QsmR. Unfortunately, however, we have not been successful in gaining meaningful results for this study yet. We have not been able to find any significant consensus sequences from our RNA-seq analysis, either. Now we are performing more rigorous analyses to identify candidate QsmR-binding sites, but we consider this research as a part of the next study following the publication of this work. We mention this point in the discussion section of the revision (lines 651 - 652 in the TC revision).

3- It should be depicted a model showing the interplay of QsmR and QS regulon on regulation of virulence factors of B. glumae strains and the points that are still undetermined indicated in dashed lines.

Response: We included a model that depicts the interplay of QsmR and the tofI/tofR QS in a new figure Fig. 12. The partial influence of QsmR on the TofI/TofR QS observed with 336gr-1 and 411gr-6 is indicated as the dashed arrows.

**Part III – Minor Issues: Editorial and Data Presentation Modifications**
Please use this section for editorial suggestions as well as relatively minor modifications of existing data that would enhance clarity.

Reviewer #1: - The pages and lines are not numbered. This made extremely difficult to refer to specific points in the text while writing the review. Please have this issue corrected during resubmission.

Response: My apology for the mistake. We numbered the pages and lines in the revision.

- Fig. 4: As far as I understand, all data presented here was normalized to wt 336, including the 411 tof mutant. A mutant should be compared to the wt of its parent strain and not to an unrelated strain. I suggest that the analysis should be split into three parts. In the first analysis, the authors should compare gene expression of 336 vs 411. In the second analysis, the tof mutant of 336 should be compared to wt 336. In the third analysis, the tof mutant of 411 should be compared to wt 411.

Response: Thank you for the good suggestion. However, we think that this chart shows the overall gene expression patterns of the major genes for toxoflavin production comprehensively, including all the three comparison points. All the four stains in the chart were assayed at the same time under same reaction condition. This chart shows the higher expression level of the genes in the highly virulent strain 411gr-6. The asterisks on the top of the data bars for 336ΔqsmR and 411ΔqsmR indicate the significant differences from the corresponding gene expression in each parent strain. This point is clarified by correction of the figure legend of Figure 4 in the revision (lines 263 – 264 in the TC revision).

- Why were the experiments conducted in Fig. 2-7 done only on 411 and 336 and not on 257? Please explain.

Response: We did not include 257sh-1 because it does not show virulence related phenotypes when this study was initiated.

- The transcriptomic analyses described in Fig. 10 for 336 seem detached from the study. I suggest the authors remove this data and publish it separately. The transcriptomic data can easily be presented in an entire standalone manuscript. Regardless, the RNA-seq data should be supplemented with an Excel sheet describing all the DEGs and fold changes, and raw data should be deposited in a public database.

Response: We are preparing another manuscript for more comprehensive analyses of transcriptome data with 336gr-1, 411gr-6 and 257sh-1, and their respective qsmR and TofIR QS mutant derivatives. This time, we present this set of transcriptome data to overview the differential regulatory functions between *qsmR* and TofIR QS. We include the DEG data in excel format as supplementary information (Supplementary Data2). We have deposited the raw RNA sequencing data, which will be released upon acceptance of this paper before publication. If the reviewer and the senior editor agree that this part of the study does not necessary, we will follow the opinion and remove the transcriptome analysis part in the second revision.

- Fig S1: Considering that a draft genome sequences are available, why did the authors opt to use a single-copy gene for the phylogenetic analysis?

Response: The tree was not created based on one gene. We used a built-in phylogenetic tree tool from BV-BRC ([https://www.bv-brc.org/app/PhylogeneticTree](https://nam02.safelinks.protection.outlook.com/?url=https%3A%2F%2Fwww.bv-brc.org%2Fapp%2FPhylogeneticTree&data=05%7C02%7CJHam%40agcenter.lsu.edu%7Ca90492c206394735935d08dc6ab1fd75%7C804b509899084bdf9c06b3df777563aa%7C0%7C0%7C638502559890404385%7CUnknown%7CTWFpbGZsb3d8eyJWIjoiMC4wLjAwMDAiLCJQIjoiV2luMzIiLCJBTiI6Ik1haWwiLCJXVCI6Mn0%3D%7C0%7C%7C%7C&sdata=PNOLQKxsn2eVRgACumwAOb5x9VBE5TyYszItHJeLfsU%3D&reserved=0)), which builds an alignment using randomly selected amino acid and nucleotide sequences from a predetermined number of the BV-BRC global Protein Families (PGFams). Single-copy genes for 1000 PGFams, the maximum number of genes allowed in the tool, were selected from the whole genome sequences of *B. gluame* strains to differentiate the closely related bacterial genomes in the phylogenetic tree.

Reviewer #2: Minor points:
-Please, clarify “lost the function” in this sentence in abstract: all the virulent strains tested lost the function almost completely upon deletion of the qsmR gene.

Response: We changed the sentence to ‘all the virulent strains tested lost the ability to produce toxoflavin almost completely upon deletion of the *qsmR* gene.’ (lines 36 – 38 in the TC revision).

-Page 10 “B. glumae 411gr-6 is a highly virulent strain that produces pigments on CPG medium, which have antimicrobial activities.” It should be highlighted in the introduction section in the MS which virulence factors in B. glumae are essential for pathogenicity. For example, protease production, T2SS, T3SS and effectors.

Response: We addressed in the introduction that ‘…toxoflavin, an azapteridine phytotoxin, is the most important virulence factor of the pathogen [3]’. To emphasize the importance of toxoflavin, we also modified a related sentence in the introduction: ‘*B. glumae* 336gr-1 is a virulent, non-pigmented strain that produces the virulence factors toxoflavin and the PrtA extracellular protease, which represent the most important and a moderately important virulence factors of *B. glumae*, respectively [10, 11, 17].’ (lines 116-119).

-Page 12 - PidS/pidR two component regulatory. Please, change “p”idR to capital letter

Response: Thank you for finding out the mistake. We corrected it.
