# Supplementary material for: qsmR encoding an IclR-family transcriptional factor is a core pathogenic determinant of Burkholderia glumae beyond the acyl-homoserine lactone-mediated quorum-sensing system
Source: PLoS Pathog. 2024 Oct 3;20(10):e1011862. doi: 10.1371/journal.ppat.1011862 (PMC11478832; doi:10.1371/journal.ppat.1011862)
Supplement: Attachment — Submitted filename: Responses-to-Reviewer.docx [file ppat.1011862.s004.docx]

**Part I - Summary**

Reviewer #1: The current submission by Lelis et al. is a revised version of the original manuscript addressing the reviewers' comments. The authors have addressed most of the issues raised by myself in the original submission.

Although the authors failed to complement the qsmR mutant, they have convinced me that qsmR is indeed a major virulence regulator by demonstrating that this phenotype occurs in multiple independently disrupted qsmR B. glumae strains. I also agree that the expression levels of transcriptional regulators like QsmR may require fine-tuning for proper function, which could explain why complementation failed.

Response: Thank you for your supportive comment on this issue.

I still think that the gene expression analysis in Figure 4 should be separated into several subfigures, each depicting the gene expression pattern in each bacterial strain.

Response: Thank you for the good suggestions. We modified Figure 4 as suggested, for which we split it into two plots for 336gr-1 and 411gr-6, respectively.

I also think that the protein expression of QsmR257 and QsmR336 should be confirmed in 257sh-1 to support the newly depicted data in Figure 9.

Response: We conducted Western blot experiments to confirm the expression of QsmR in *B. glumae*, using anti-QsmR polyclonal antibodies raised from rabbit. We could detect both QsmR_336_ and QsmR_257_ expressed in *B. glumae* 257sh-1 from the respective *qsmR* clones. This result is shown as Figure 9E and the procedures for the westernblot experiments are added to the Materials and Methods section in the revised manuscript (lines 857 – 875 in the revision with track change (TC)).

Reviewer #2: The authors have addressed most of the queries in revised manuscript and added changes in text which helped to clarify some of the points indicated by the reviewers. I really miss the molecular mechanism by which QsmR can contribute in regulating the virulence factors in Burkholderia glumae, though I suppose that it should be very complex mechanism and may related with fine-tuning expression of qsmR itself and of the factors under its regulation. The findings described in the work are interesting and provoke for searching a better understanding of QmsR regulation in future researches. The findings described in the work are interesting and may help to enforce food security by chasing a inhibitor of QsmR. I recommend this revised work for publication.

Response: We appreciate your supportive comments on the revised manuscript.

**Part II – Major Issues: Key Experiments Required for Acceptance**

Reviewer #1: (No Response)

Reviewer #2: (No Response)

**Part III – Minor Issues: Editorial and Data Presentation Modifications**

Reviewer #1: 1. Figure 4: As I mentioned in the first revision cycle, I think that each mutant should be compared to its parent strain and not collectively compared to strain 336. Please reanalyze the data as requested previously and split the figure into three subfigures.

Response: As mentioned above, we modified Figure 4 following this suggestion.

2. Figure 9: I think that the newly depicted data in Figures 9A and 9B support that variation in QsmR is a significant contributing factor to the variation in virulence identified between the strains. However, I think this data would be more convincing if complementation is supported by a western blot. If the authors demonstrate that, despite containing the plasmid-encoded qsmR variants, there is a clear difference in the protein accumulation between QsmR257 and QsmR336, the data would still be valid and could be a point for discussion since sequence variation might affect protein stability or RNA structure.

Response: As mentioned above, we conducted western blot to demonstrate the expression of QsmR_257_ and QsmR_336_ in 257sh-1 carrying plasmid for heterologous expression of each protein. This result is presented as Figure 9E, in which QsmR_257_ and QsmR_336_ were also detected in the wild type strains of 336gr-1 and 257sh-1 as less amounts but not in their *ΔqsmR* derivatives (336ΔqsmR and 257ΔqsmR). This western blot result indicates that the lost function of *qsmR_257_* is not due to the unstable RNA or protein. We address this western blot test in the Materials and Methods (lines 857 – 875), Results (lines 399 – 400), Figure Legend (lines 418 – 426), and Discussion (lines 549 – 553) sections of the revision with TC.

3. Table 1: Can you add another "WT" column in addition to ΔtofI and ΔqsmR?

Response: Thank you for the good suggestion. Table 1 has been modified as suggested.

4. I see according to the figure legends that while most experiments were repeated at least twice, some of the rice inoculation experiments were repeated only once (Figures 6C, 6D, 9C, and 9D). Is this indeed the case? If so, what was the reason that the authors did not repeat these particular experiments?

Response: The virulence tests with rice plants require precise timing of inoculation within a narrow growth stage window and optimal environmental conditions for pathogen infection, even under greenhouse conditions. Additionally, accidental infections by other pathogens often occur, further complicating the inoculation process. As a result, obtaining complete datasets with appropriate replications is highly challenging. This is indeed the main reason why we developed a surrogate virulence assay system with onion bulb scales (Karki et al., 2012, PLOS One). Although the datasets presented in Figures 6 and 9 come from a single experiment, we consider them reliable, as the individual mutants have been tested multiple times in previous experiments, consistenly yielding similar results.

5. Regarding the incorporation of the RNA seq analysis, I suggested doing so because I feel that the authors would benefit if these results were more highlighted in a standalone independent manuscript that would place more emphasis on the transcriptome data. However, the authors can choose to keep the data as part of the manuscript if they see fit.

Response: Thank you for the thoughtful suggestion. Although this is a great suggestion, we would like to keep this data for this manuscript to avoid major modification of the manuscript. We are working on more complehensive RNA-seq analyses, as well as ChIP-seq analyses, with new sets of samples for another manuscript focusing on genomewide regulons of QS and *qsmR* in differnet *B. glumae* strains.

Reviewer #2: (No Response)
